# Viral protease sensing for programmable antiviral lytic cell death: The path to trials

**DOI:** 10.1002/ctm2.70842

**Published:** 2026-09-16

**Authors:** Lin Li, Xiu‐Li Yan, Cheng‐Feng Qin

**Affiliations:** ^1^ State Key Laboratory of Pathogen and Biosecurity Academy of Military Medical Sciences Beijing China

1

Antiviral pharmacology has largely been built around a straightforward principle: interrupt an essential step in the viral life cycle. Entry and fusion inhibitors block access to susceptible cells, polymerase and reverse transcriptase inhibitors suppress genome replication, and protease inhibitors disrupt viral protein processing. Despite acting at different stages, these therapies largely share a common logic, blocking a function required for productive infection.^1^ Could a virus‐encoded activity instead be exploited as a signal that identifies an infected cell and initiates a therapeutic response?

In our recent study published in *Cell*, we explored this possibility by developing viral protease‐initiated lytic cell death (VID) and corresponding VID activators (VIDAs).^2^ The system exploits the modular architecture of gasdermin D (GSDMD), in which a pore‐forming N‐terminal domain is restrained by an autoinhibitory C‐terminal domain until proteolytic cleavage releases lytic activity.^3^ By engineering viral protease‐recognition sequences into GSDMD, we converted viral proteolytic activity into a trigger for infected‐cell elimination. Delivered as mRNA formulated in lipid nanoparticles (LNPs), VIDA suppressed hepatitis A virus replication and shedding and mitigated liver injury in mice, while the same design principle was extended to Zika virus and severe acute respiratory syndrome coronavirus 2 (SARS‑CoV‑2).^2^

Conventional antivirals generally work by blocking processes required for viral infection or replication. VIDA follows a different logic: rather than blocking a viral activity, it uses that activity as a signal to activate a therapeutic response. In our implementation, viral protease activity provides that signal: the same enzyme that processes viral substrates cleaves engineered GSDMD, thereby initiating lytic cell death. This creates a sense‐and‐respond system in which viral protease activity directly triggers lytic elimination of the infected cell. Therapeutic mRNA may reach both infected and uninfected cells, whereas effector activation is intended to occur preferentially where the cognate viral protease is active (Figure 1).

**FIGURE 1:**
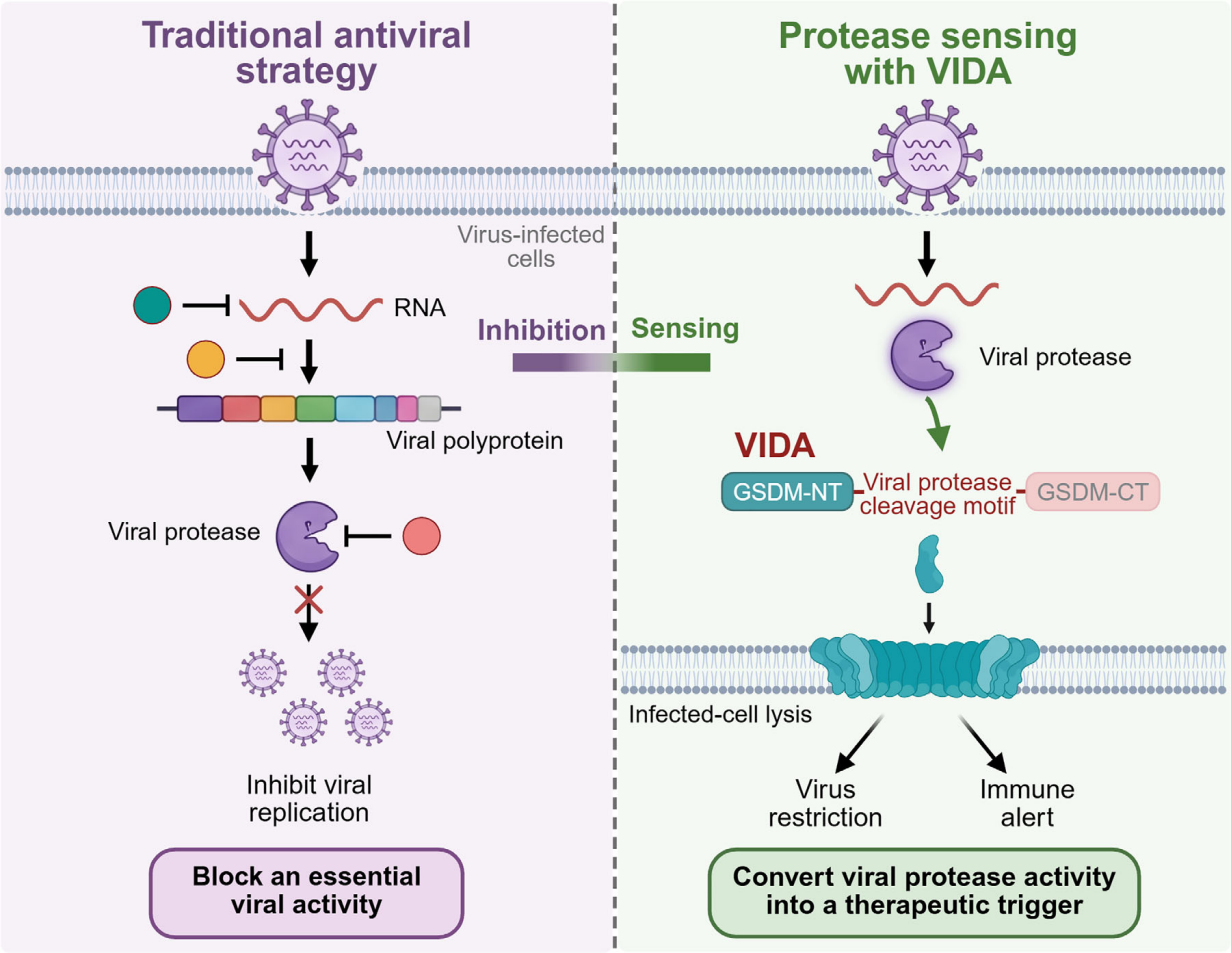
From conventional antiviral inhibition to protease sensing with VIDA. Conventional direct‐acting antivirals suppress viral replication by targeting essential viral processes, such as viral RNA synthesis and protease‐mediated polyprotein processing. Viral protease‐initiated lytic cell death activator (VIDA) instead repurposes virus‐encoded protease activity as an infection‐specific therapeutic trigger. Protease‐mediated cleavage of engineered gasdermin (GSDM) releases its pore‐forming N‐terminal domain, inducing lytic elimination of infected cells and a concomitant local immune alert. This strategy therefore shifts viral protease activity from a target for inhibition to a signal that drives a coordinated “kill‐and‐alert” antiviral response.

The choice of cell death as the therapeutic output is consequential. Rather than attempting to suppress every round of intracellular replication, VIDA removes the infected cell itself as a site of virus production. Programmed lytic cell death therefore acts as an antiviral effector that terminates a cellular niche supporting replication.^4^ VIDA thus couples pathogen‐specific sensing to deliberate control of infected‐cell fate.

The lytic response may also do more than remove virus‐producing cells. In the HAV model, VIDA treatment was associated with chemokine induction and broader antiviral immune activation without a parallel increase in several major inflammatory cytokines, consistent with a potential “kill‐and‐alert” effect.^2^ Such immune signalling could help control residual infection, but it also marks an important safety boundary. Its consequences are likely to vary with viral burden, treatment timing, tissue context, dose and the proportion of cells undergoing lysis.

One question raised by this strategy is how viral escape might differ from resistance to conventional protease inhibitors. Inhibitor resistance can arise when mutations reduce drug binding while preserving or restoring sufficient catalytic activity.^5^, ^6^ Escape from a protease‐responsive sensor could instead involve changes in substrate recognition that must remain compatible with processing of essential viral substrates. This constraint should not be overstated: altered substrate preference, cleavage kinetics, protease abundance or sensor accessibility could all reduce VIDA activation. Whether multi‐motif or multi‐protease designs can make the system more robust will need to be tested experimentally.

Retargeting VIDA to another virus will likewise require more than simply replacing a cleavage motif. In our study, generative artificial intelligence was used to explore the sequence space of SARS‐CoV‐2 main protease cleavage motifs and identify optimised VIDA variants.^2^ Computational approaches may accelerate this step, but successful retargeting will still depend on protease biology, substrate accessibility, and the properties of the infected tissue.

These considerations suggest a practical way to think about translation: which virus, which tissue, and which disease stage. A suitable virus should encode a protease that is sufficiently active and accessible to support reliable activation, with substrate‐recognition features sufficiently conserved across clinically relevant strains. The target tissue must also be reachable by the delivery system and able to tolerate selective loss of infected cells. Systemically administered LNPs preferentially deliver to the liver, whereas efficient delivery to many extrahepatic tissues remains challenging.^7^, ^8^ Even successful delivery, however, does not ensure an acceptable therapeutic window. A strategy tolerated in hepatocytes or renewable epithelia cannot automatically be extrapolated to neurons, cardiomyocytes or other cell populations with limited regenerative capacity.

Disease stage is equally important. Patients treated while active viral replication remains a major driver of pathology may have a wider therapeutic window than those in whom extensive tissue injury or immune‐mediated pathology has already developed. Infection‐triggered cell elimination is therefore unlikely to have the same benefit‐risk profile throughout the course of disease.

Initial clinical development may be most informative in acute infections with measurable viral kinetics, a well‐defined protease trigger, an accessible target tissue, and sufficient organ reserve to tolerate selective infected‐cell loss. Early studies should examine not only viral load or shedding, but also infection‐dependent activation, preservation of organ function, and systemic and tissue inflammatory responses. The most suitable indications will therefore be those in which protease activity, tissue accessibility, and tolerance of infected‐cell elimination overlap within a clinically useful window.

## AUTHOR CONTRIBUTIONS

L.L. drafted the manuscript. X.‐L.Y. reviewed and edited the manuscript. C.‐F.Q. supervised the work and critically revised the manuscript. All authors approved the final manuscript.

## CONFLICT OF INTEREST STATEMENT

The authors have filed patents related to VIDA.

## ETHICS STATEMENT

Not applicable. This article does not report new studies involving human participants or animals.

## Data Availability

Data sharing is not applicable to this article as no new data were created or analyzed in this study.
